# Novel Therapies for the Treatment of Neuropathic Pain: Potential and Pitfalls

**DOI:** 10.3390/jcm11113002

**Published:** 2022-05-26

**Authors:** Pottathil Shinu, Mohamed A. Morsy, Anroop B. Nair, Abdulaziz K. Al Mouslem, Katharigatta N. Venugopala, Manoj Goyal, Monika Bansal, Shery Jacob, Pran Kishore Deb

**Affiliations:** 1 Department of Biomedical Sciences, College of Clinical Pharmacy, King Faisal University, Al-Ahsa 31982, Saudi Arabia; 2 Department of Pharmaceutical Sciences, College of Clinical Pharmacy, King Faisal University, Al-Ahsa 31982, Saudi Arabia; momorsy@kfu.edu.sa (M.A.M.); anair@kfu.edu.sa (A.B.N.); aalmoslem@kfu.edu.sa (A.K.A.M.); kvenugopala@kfu.edu.sa (K.N.V.); 3 Department of Pharmacology, Faculty of Medicine, Minia University, El-Minia 61511, Egypt; 4 Department of Biotechnology and Food Science, Faculty of Applied Sciences, Durban University of Technology, Durban 4000, South Africa; 5 Department of Anesthesia Technology, College of Applied Medical Sciences in Jubail, Imam Abdul Rahman Bin Faisal University, Jubail 35816, Saudi Arabia; mggoyal@iau.edu.sa; 6 Department of Neuroscience Technology, College of Applied Medical Sciences in Jubail, Imam Abdul Rahman Bin Faisal University, Jubail 35816, Saudi Arabia; mbbanasl@iau.edu.sa; 7 Department of Pharmaceutical Sciences, College of Pharmacy, Gulf Medical University, Ajman 4184, United Arab Emirates; sheryjacob6876@gmail.com; 8 Department of Pharmaceutical Sciences, Faculty of Pharmacy, Philadelphia University, Amman 19392, Jordan; prankishore1@gmail.com

**Keywords:** neuropathic pain, spinal cord stimulation, transcranial magnetic stimulation

## Abstract

Neuropathic pain affects more than one million people across the globe. The quality of life of people suffering from neuropathic pain has been considerably declining due to the unavailability of appropriate therapeutics. Currently, available treatment options can only treat patients symptomatically, but they are associated with severe adverse side effects and the development of tolerance over prolonged use. In the past decade, researchers were able to gain a better understanding of the mechanisms involved in neuropathic pain; thus, continuous efforts are evident, aiming to develop novel interventions with better efficacy instead of symptomatic treatment. The current review discusses the latest interventional strategies used in the treatment and management of neuropathic pain. This review also provides insights into the present scenario of pain research, particularly various interventional techniques such as spinal cord stimulation, steroid injection, neural blockade, transcranial/epidural stimulation, deep brain stimulation, percutaneous electrical nerve stimulation, neuroablative procedures, opto/chemogenetics, gene therapy, etc. In a nutshell, most of the above techniques are at preclinical stage and facing difficulty in translation to clinical studies due to the non-availability of appropriate methodologies. Therefore, continuing research on these interventional strategies may help in the development of promising novel therapies that can improve the quality of life of patients suffering from neuropathic pain.

## 1. Introduction

The Neuropathic Pain Special Interest Group (NeuPSIG) of the International Association for the Study of Pain (IASP) has given the most widely accepted definition of neuropathic pain as “pain caused by a lesion or disease of the primary afferent neurons of the somatosensory nervous system”, which includes peripheral neuropathy, postherpetic neuralgia (PHN), trigeminal neuralgia, nerve root pain, and phantom limb pain [1]. In general, somatosensory nerves originate in the skin, joints, muscles, and fascia, and are involved in the perceptions of touch, vibration, position, movement, temperature, pressure, and pain. Lesions or diseases of these nerves may lead to altered sensory transmission to the spinal cord or the brain, resulting in neuropathic pain [2]. Not all individuals with central nervous system injury or peripheral neuropathy tend to develop neuropathic pain [3]. Neuropathic pain represents a mechanistic dissimilarity from other chronic pains requiring different diagnosis and treatment strategies [4]. Global epidemiologic surveys have shown that the incidence of chronic neuropathic pain is 10–14% in the United Kingdom [5], 9.8–12.4% in the United States [6], and 7–10% worldwide [2]. It is more frequent in women than men and has a higher prevalence in the age group above fifty years. Moreover, chronic neuropathic pain is one of the major reasons for medical consultations among Americans. As per the International Classification of Diseases and Related Health Problems 11 (ICD11), neuropathic pain can mainly be categorized as peripheral (including post-amputation pain, trigeminal neuralgia, PHN, painful radiculopathy, painful polyneuropathy, and peripheral nerve injury pain) and central (including central post-stroke pain, spinal cord or brain injury neuropathic pain, and central pain in multiple sclerosis) neuropathic pain [7]. Usually, patients complain about intermittent or ongoing spontaneous pain, described as pricking, shooting, squeezing, burning, freezing, or sometimes, paroxysmal electric-shock-like pain. Non-painful altered sensations (e.g., dysesthesia and paresthesia), evoked pains including allodynia and hyperalgesia, and after-sensations (such as referred pain and hyperpathia), in addition to spontaneous pain (which rarely occurs as a single manifestation), may be reported [8]. Nevertheless, poor associations and discrepancies related to bedside findings and quantitative sensory testing remain a major issue. The three types of chronic pain include (i) neuropathic pain, caused by an abnormality in the somatosensory system (for example, diabetic neuropathy), (ii) nociceptive pain due to tissue damage (for instance, osteoarthritis), and (iii) mixed pain, in which both nociceptive and neuropathic pains exist, such as in chronic radicular back pain [9]. Research for therapies of persistent neuropathic pain is ongoing, because systemic analgesic drugs have failed due to a lack of target specificity and the development of tolerance [10]. Although several clinical trials on different pharmacologic interventions for neuropathic pain have been conducted in recent years, most of them either argued about the consistencies of existing clinical evidence or concluded with discordant summaries due to methodological variations in evidence assessments [11]. Ultimately, poor pharmacologic outcomes have led to the recommendation of non-pharmacologic interventions (such as surgery), which are known for their partial/inadequate pain relief or intolerable adverse reactions [12]. Despite these challenges, there have been various developments in comprehending the pathophysiology of pain and developing new approaches for diagnoses, which play an important role in creating advanced interventional therapies. Furthermore, contemporary pain management therapies only focus on treating the symptoms of pain, not treating the actual cause of it. Therefore, in this review article, we explore the pain pathway with the aim of analyzing both clinical intervention strategies and preclinical future directions in the treatment and management of neuropathic pain. The current pharmacological agents for neuropathic pain, such as gabapentinoids, lidocaine, serotonin and norepinephrine reuptake inhibitors (SNRIs), etc., are the most popularly used medication, but have major drawbacks because of their CNS toxicity. Furthermore, due to long-term treatment such as in cases of chronic pain, patients might fail to respond to conservative therapy. Therefore, for such patients, interventional approaches become the most reasonable alternatives for pain management. Thus, this review will also provide some insights into the present scenario in the pain research field, particularly various interventional techniques such as spinal cord stimulation, steroid injection and neural blockade, transcranial/epidural stimulation, deep brain stimulation, percutaneous electrical nerve stimulation, neuroablative procedures, opto/chemogenetics, gene therapy, and ion channel targeting.

## 2. Selection of the Literature

**Figure 1:**
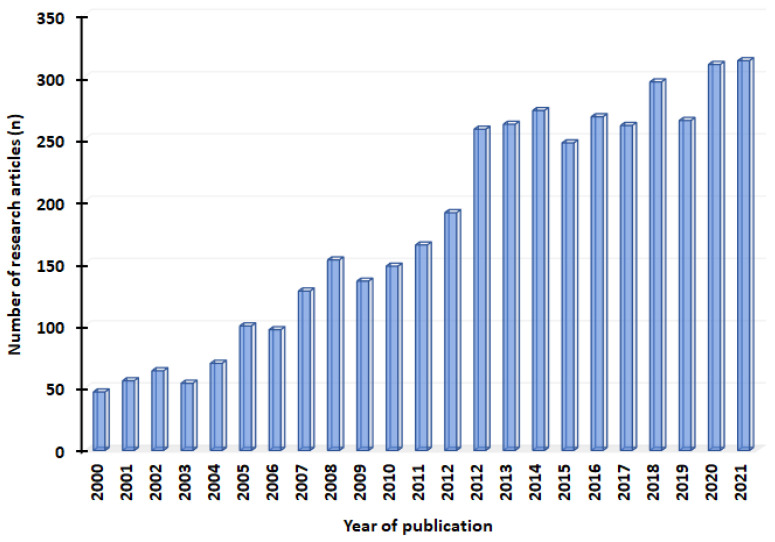
The number of articles published in the treatment of neuropathic pain from 2000 to 2021.

To search for suitable articles, we used keywords such as “spinal cord injection”, “steroid injection”, neural blockade”, “transcranial /epidural stimulation”, “percutaneous electrical nerve stimulation”, “neuroablative procedures”, “percutaneous electrical nerve stimulation” “deep brain stimulation”, “chemo genetics”, and “ion channel targeting”. All the keywords used to search the articles were used either alone or in combination. Out of the N = 4162 articles published from 2000 to 2021, a total of N = 160 potentially relevant articles were included for final analysis. Most of the manuscripts were excluded after reading the title and abstract. We selected manuscripts published in the English language and excluded nonneuropathic pain literature or manuscripts with expert opinions. However, several manuscripts were excluded after reading the full texts. This was mainly due to the lack of a clear description of the type of pain management used in the study or due to the absence of its outcome. Furthermore, the literature that provided conservative pain management techniques and referrals to other studies were also excluded from the current review. Figure 1 depicts the details of neuropathic pain-related articles published every year from 2000 to 2021.

## 3. Pathophysiology of Neuropathic Pain

Neuropathic pain results from diverse pathobiological mechanisms, which were mainly perceived by studying the experimental animal models. Among these diverse mechanisms, two of them were predominantly observed, i.e., peripheral and central sensitization [9]. Other mechanisms included the infiltration of activated macrophages and release of pro-inflammatory cytokines, central disinhibition, uncontrolled modulation caused by decreased inhibitory neurotransmitters (gamma-aminobutyric acid (GABA), glycine), genetic modifications (e.g., SCN9A gene) [13], microglial activation, anatomical reorganization of the spinal cord, and abnormality in the somatosensory cortex [14].

As the pathological process progresses, there are increases in the nitric oxide synthase levels of the axotomized neurons, increases in the sensitivity of spinal neurons after cell death, long-lasting signal transmissions of the spinal synapse, and diminished central pain inhibitory mechanisms [14]. In addition, the pathological changes at the cellular level include apoptosis, which might induce neuronal sensitization, altering inhibitory systems, which could eventually cause brain damage, ischemia, or neuropathic pain [15].

**Figure 2:**
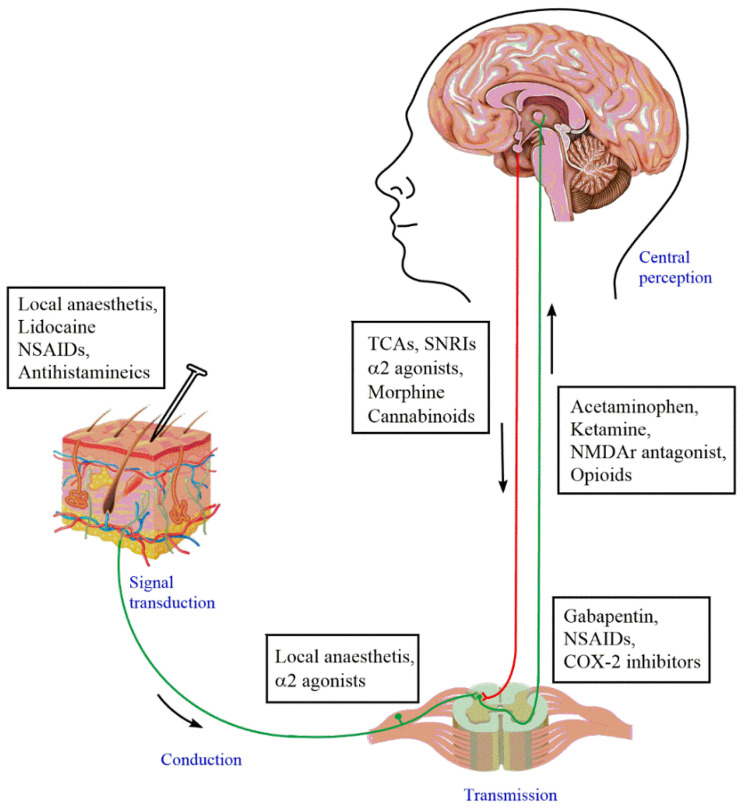
Pain pathway and pharmacological interventions. From tissue trauma to central sensitization, different analgesics are known to act at various points of pain transmission. At the site of injury, local anaesthetics, antihistamines, and anti-inflammatory agents can be used for direct pain relief. Opioids and non-opioid drugs including morphine, cannabinoids, COX-2 inhibitors, α2 agonists, gabapentin, acetaminophen, and tricyclic anti-depressants (TCAs) act both peripherally as well as centrally, hence attenuating the transmission of pain signaling.

Upon injury or any kind of damage, the first-order neurons, also known as nociceptors, are activated first. These nociceptors are responsible for the transduction of pain signals. These signals are then conducted and transmitted to the dorsal horn of the spinal cord. In the spinal cord, the second-order neurons in the cell body are neurons found in the Rexed laminae of the spinal cord. The dorsal horn of the spinal cord consists of the synaptic integration of first-order and second-order neurons, along with the inter neurons. Furthermore, from here, the second-order neurons ascend towards the relay center of the brain, which is the ventral posterolateral nucleus of the thalamus via the spinothalamic tract. From the thalamus, the cell bodies of the third-order neurons carry the signals to the somatosensory cortex. In physiological conditions, pain could cease by the activation of the descending pain pathway, but in pathological conditions, this pathway may become hampered. Therefore, for the effective treatment of pain, the transmission of pain signals can be attenuated at various points to reduce the firing of the action potentials. Contemporary pharmacological interventions also focus on targeting different sites of the pain pathway, as shown in Figure 2.

### 3.1. Peripheral Sensitization

Peripheral sensitization is caused by non-myelinated C-fibers as well as thin Aδ afferent neurons. These mainly respond to noxious stimuli such as a lesion, injury, or disease. As a result of such stimuli, these neurons become abnormally sensitive and can be studied by the major changes that occur at the molecular or cellular levels [16]. The primary afferent neurons’ hyper-responsiveness causes the initiation of ectopic spontaneous activity via the voltage-gated sodium channels, particularly through Na_v_1.7, Na_v_1.8, along with Na_v_1.9 [17]. These changes are observed not only at the injured site, but also around the nearest intact dorsal root ganglia (DRG). This theory has been proven indirectly by the decrease in pain using the non-selective sodium channel blocker lidocaine by effectively blocking the NaV channels [18]. Among these voltage ion channels, Na_v_1.7 has been implicated to have a vital role in the generation of pain signals, as revealed by a mounting number of genetic studies both pre-clinically and clinically [19,20]. However, at present, the possible extent of Na_v_1.7 inhibition required for eliciting the desired analgesic effect, eliminating the pathological pain while maintaining normal nociceptive functioning, is unclear. Furthermore, Na_v_1.7-mediated analgesia from the peripheral sensory nerve endings to the spinal projections has been found to be of potential therapeutic interest [21]. Na_v_1.8 has also been implicated to contribute to the development of increased noxious as well as non-noxious stimuli. Recent studies on Na_v_1.8 knockout animals have also demonstrated this channel to be involved in the pathogenesis and maintenance of neuropathic pain by contributing to the development of allodynia and hyperalgesia [22]. Similarly, Na_v_1.9 has also been found to be a crucial mediator of visceral pain pathways, and its mutation has been associated with insensitivity to pain [23]. Another interesting observation was noted in the transient receptor potential cation channel subfamily V member 1 expression (which usually responds to thermal stimuli above 43 °C). However, after partial injury, there is a decreased expression of these receptors, unlike what is expected to show upregulation [17].

### 3.2. Central Sensitization

Modification of the spinal cord results in central sensitization. Due to peripheral sensitization, there is an increased reactivity of C-fibers to the stimuli, which causes the release of glutamate that acts on the N-methyl-D-aspartate (NMDA) receptors, showing central sensitization. At the same time, there is an increase in the release of substance P, which also facilitates central sensitization. In such conditions, the normal innocuous stimuli will start activating the spinal cord neurons (Aδ and Aβ). To control these irregular activities, there must be a release of the inhibitory neurotransmitter GABA by the interneurons. However, they were found to be damaged in the case of rodent models as a result of the injury that leads to further central sensitization [17]. Other types of synaptic plasticity at the spinal cord or supraspinal levels induced by noxious stimuli modulate nociceptive transmission, which includes an increase in the trafficking of ion channels and receptors to the membrane, altered function by phosphorylation, and gene transcription [24,25,26]. Transcription-independent phenomena include windup and long-term potentiation, whereas a transcription-dependent phenomenon is long-lasting facilitation [25]. A detailed discussion on the pathophysiology of neuropathic pain is out of the scope of this article; however, this is described elsewhere in the literature [8,16,27].

## 4. Clinical Symptoms in Patients with Neuropathic Pain

**Figure 3:**
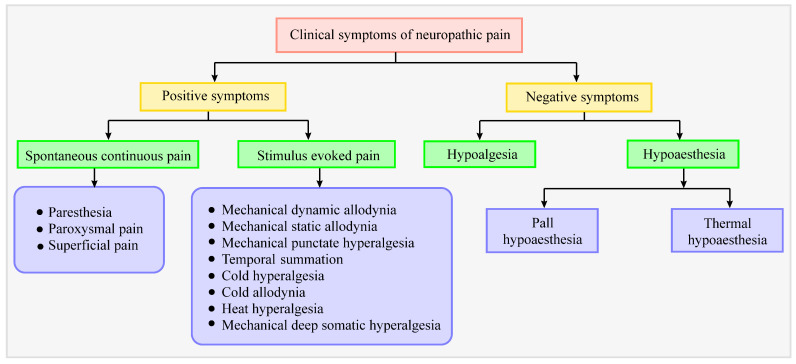
Flowchart depicting positive and negative symptoms of neuropathic pain.

Assessment of the clinical symptoms of neuropathic pain will help to obtain better insights into the mechanisms involved and to identify novel therapeutic approaches. The basis of neuropathic pain is lesion or injury to afferent neurons, which leads to incomplete inputs to the nervous system, resulting in sensory loss and developing negative sensory symptoms. A flow chart depicting positive and negative symptoms of neuropathic pain is presented in Figure 3. Positive sensory symptoms are due to hyperactivity and increased sensitivity in nociceptors, which are characterized by two hallmark features; allodynia and hyperalgesia [28]. Sensory deficits usually include mechanical and thermal hypersensitivity. Positive (gain-of-function) symptoms include paresthesia (skin-crawling sensation or tingling), electric-shock-like sensations, spontaneous (not induced by a stimulus) ongoing pain, as well as shooting pain [29], whereas the negative (loss-of-function) symptoms include hypoalgesia (decreased sensitivity to nociceptive stimulus), hypoesthesia (reduced sensitivity to vibrations, numbness), weakness, and reflex changes [30]. In addition, most of the patients have hypersensitivity, burning, smarting, lancinating, and shock-like pains [31,32].

## 5. Diagnosis of Neuropathic Pain

Accurate diagnosis is a critical step to providing proper treatment for any disability. However, the differentiation between neuropathic and nociceptive pain becomes difficult, because they are closely linked via several pain pathways [33]. Performing proper diagnoses depends on a few aspects, such as medical history and physical examination. Electrophysiological, histological, and structural imaging tests are performed to confirm the diagnosis [34]. Electrophysiological techniques are crucial to identify any somatosensory lesions due to an injury or disease by the use of electrical stimulations on large and small fibers together to determine the nerve conduction velocity. Moreover, nociceptive evoked potentials at adequate sensitivity and intensity are used to identify any damage to the nerve. Imagining techniques such as MRI and ultrasound, along with echotexture, are also used for the early visualization and diagnosis of peripheral neuropathy [35]. Pain is considered to be an individual’s experience with varying symptoms among different patients. In most cases, neuropathic pain has been understood as a combination of positive and negative symptoms. A single symptom cannot predict neuropathic pain, but certain symptoms, along with bedside findings and pain descriptors, elevate the probability of identifying a neuropathic pain-like state.

Pain is subjective and has no validated diagnostic criteria, making it difficult for clinicians to recognize it. However, four main diagnostic tools are used: (i) medical history, (ii) clinical examinations, (iii) laboratory testing, and (iv) functional imaging tools [16,36]. Abnormal sensation or hypersensitivity can often be observed, which could be in the affected area or areas surrounding the affected area. Additional assessments are usually performed by pricking, touching, or applying thermal stimuli (cold and hot) or pressure.

Other than these methods, there are other diagnosis methods which rely on the patient’s expression of the condition: verbal screening tools. Several questionnaires have been designed to identify neuropathic pain. These include PainDETECT, ID-Pain, Neuropathic Pain Questionnaires, Neuropathic Pain Symptom Inventory, Leeds Assessment of Neuropathic Symptoms and Signs, and Douleur Neuropathique 4 questions [2,32]. The questions are designed to find out about pain attacks, tactile and thermal hypersensitivity, tingling, prickling, and insensibility. Imaging techniques such as computed tomography (CT scan), positron emission tomography (PET scan), and magnetic resonance imaging (MRI) are performed in case of a requirement to detect nerve compression and nerve infiltration [37]. Various laboratory tests need to be performed on certain neuropathic pain conditions, including microneurography (a minimally invasive technique to record nerve fiber activity), nerve biopsy, skin biopsy, and punch biopsy [38,39]. The pain scales and details of the symptoms are crucial in evaluating the effects of any new therapy or the efficacy of a new molecule.

## 6. Current Status of Pharmacotherapies for Neuropathic Pain: Advantages and Disadvantages

Current evidence indicates that non-pharmacological approaches could be reasonable to eliminate or reduce the necessity for potentially noxious medicaments and enhance analgesic regimens’ efficacy [40]. The recommended first-line drugs in neuropathic pain management include tricyclic antidepressants, SNRIs, gabapentin, and pregabalin [41]. Tricyclic antidepressants (mainly amitriptyline) are the primary treatment option for neuropathic pain, but can induce cognitive impairment or turmoil, walking disturbance, and falls, especially in old-aged patients at risk of suicide or death due to accidental overdose [42]. It is better to avoid such drugs in patients suffering from cardiovascular disease, because this may result in decreased blood flow to the heart or high vulnerability to sudden cardiac arrest [43]. FDA-approved drugs that block the reuptake of serotonin and norepinephrine (namely, SNRIs), such as duloxetine and venlafaxine, are used in the therapy of peripheral diabetic neuropathy, fibromyalgia, and back pain, but have potential side effects. However, clinicians do not prescribe venlafaxine because of its withdrawal syndrome [44,45]. Gabapentin and pregabalin are other first-line drugs used in the management of diabetes-induced peripheral neuropathy and PHN. Gabapentin was approved for the treatment of PHN due to its increased affinity towards the α2-δ subunit of voltage-gated calcium channels that cause a reduction in calcium influx in the neuron and successive neurotransmitters [46]. However, it is ineffective in chemotherapy-induced painful neuropathy. Vertigo, hypersomnia, peripheral edema, obesity, and xerostomia are the most common adverse effects seen in patients administered gabapentin [47].

Second-line drugs in treating neuropathic pain include topical agents such as lidocaine, capsaicin, and tramadol (a weak opioid and SNRI). Patches containing 5% lidocaine have been assessed for the treatment of peripheral neuropathic pain, especially PHN. However, the therapeutic potential was modest compared with the placebo. Side effects are limited to applied areas and may cause skin irritation [41]. Capsaicin (TRPV1 modulator) in the form of patches (8%) is known to demonstrate sustained efficacy (~3 months) in diabetic or non-diabetic neuropathies. Systemic absorption is minimal, and adverse effects include transient skin reactions such as pain, itching, and redness. Although treatment can be repeated after three months, there are no long-term data available on the effect on nerve fibers [41,48]. Opioid weak agonists such as tramadol (which also acts as an SNRI) have been proven to be efficient in patients with neuropathic pain [49]. This drug has a lower risk of abuse than strong opioids, but possesses a risk of somnolence and confusion in adults [50].

Third-line treatments include certain strong opioids such as morphine and oxycodone, which are moderately helpful in peripheral neuropathic pain. However, opioid-phobia seems to have increased among people due to the likelihood of the misuse of the drugs and addiction potential, which lead to a reduction in opioid use [41,51,52]. Subcutaneous botulinum toxin type A (a neurotoxin) is also recommended as a third-line therapy or as the final option in refractory cases. Application of the drug is painful, but evidence of long-term effects is limited [53].

There are a plethora of molecules and compounds that researchers are working on to find an effective pain treatment. Flavonoids are such compounds with a safe pre-clinical profile. These phytoconstituents are known to have anti-inflammatory and antioxidant properties, which are further being exploited to find a suitable cure for painful conditions. Flavonoids are currently widely popular due to their minimal CNS toxicity, as shown in animal models of pain. Moreover, it is found that flavonoids not only exhibit analgesic properties by inhibiting and preventing the synthesis of COX-2, but also reduce the side effects caused by NSAIDs [54]. Although this metabolite has shown tremendous potential for relieving pain effectively, the results are still limited to pre-clinical studies, lacking sufficient clinical evidence of its therapeutic effects.

Apart from these, several drugs or classes of drugs have demonstrated inconsistent results, including tapentadol (opioid agonist), cannabinoids, NMDA antagonists, selective serotonin reuptake inhibitors (SSRIs), etc. Due to ineffective analgesic agents for chronic pain, only adequate pharmacological treatment options for symptoms are currently available. Table 1 summarizes the currently used front-line drugs for treating neuropathic pain along with their limitations.

## 7. Interventional Methods as an Effective Treatment Approach against Neuropathic Pain

Available pharmacotherapeutics are unable to manage pain on a long-term basis and patients are developing resistance and tolerance to pain management medications over time; therefore, it is necessary to investigate non-pharmacological or interventional approaches. Interventional treatments, including nerve blocks or surgeries for targeted drug delivery, or specific neural modulation, provide alternate strategies in refractory cases [12,56]. In this review, we classified the currently available treatment approaches into two broad categories, clinical interventional techniques and preclinical or novel experimental pain-controlling agents. Clinical interventional techniques are those techniques that are currently practiced or used in human pain management. These techniques mainly include spinal cord stimulation (SCS), steroid injection/neural blockade, transcranial/epidural stimulation, deep brain stimulation (DBS), percutaneous electrical nerve stimulation (PENS), and neuroablative procedures. The techniques that are under development or not used in human pain management are classified as preclinical or novel experimental pain-controlling agents. These techniques include opto/chemogenetics, gene therapy, and ion channel targeting.

### 7.1. Clinical Interventional Techniques

#### 7.1.1. Spinal Cord Stimulation (SCS)

Based on gate-control theory, the stimulation of large myelinated Aβ-fibers with low-intensity electrical signals was introduced to modulate the pain signals conducted by the unmyelinated C-fibers [2]. SCS is a well-established technique among other neuromodulatory methods. It acts upon nerves and alters nerve activity by providing electrical stimuli to the target site [57]. SCS is a clinical application arising directly from the revolutionary “gate-control” theory of pain suggested by Melzack and Wall [58]. This theory hypothesized the concept of an intrinsic pain control mechanism [59]. For SCS therapy, a series of electrodes (4–16) is placed in the epidural spaces partly covering the dorsal columns of the vertebra; the dorsal column should innervate the painful area as well [60]. The low-voltage electrical current in the nerve fibers of the spinal cord is supplied by a pulse generator which is directly connected to a power source. The electrical stimulation thus generated can have both an inhibitory effect and stimulatory effect on pain signals and nerve impulses, respectively [61].

**Figure 4:**
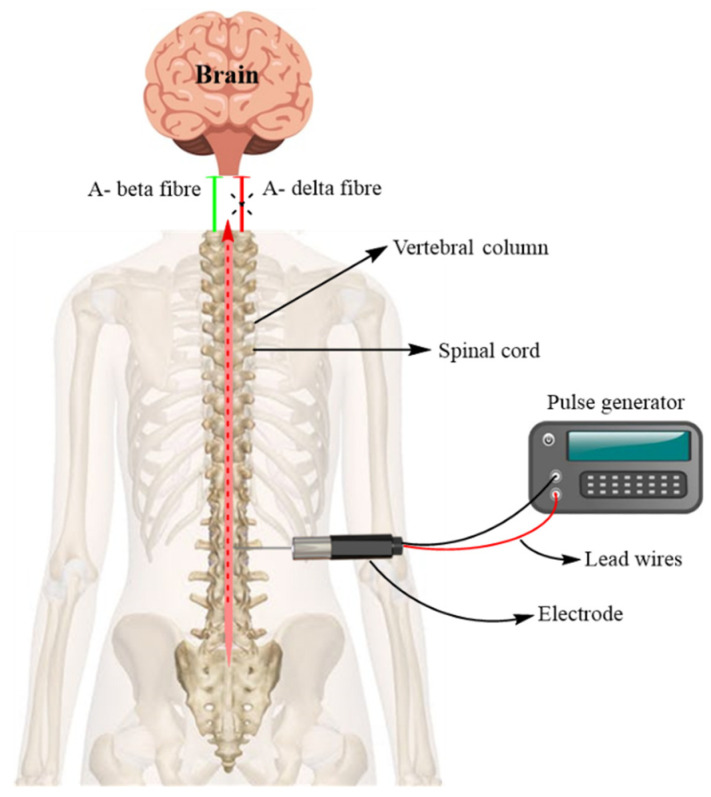
Spinal cord stimulation therapy for neuropathic pain. A pulse generator sends a low-threshold electric current through an extension wire to an electrode placed in the epidural space. Generated non-nociceptive signals (Aβ fibers) mask the travel of nociceptive signals (Aδ and C-fibers) to the brain, thus causing pain relief.

The attenuation of neuropathic pain by stimulating the spinal cord involves the inhibition of nociceptive input carried by thinly myelinated or unmyelinated fibers (Aδ as well as C-fibers), and interruption in the transmission of nociceptive signals to the brain [62]. A schematic representation of SCS therapy for neuropathic pain is depicted in Figure 4. The neurochemical mechanism comprises the release of various neurotransmitters involved in pain attenuation, such as GABA, serotonin, acetylcholine, and norepinephrine [63]. In the process of SCS, neurotransmitters may stimulate spinal GABAergic circuitry receptors located on GABAergic interneurons present in the spinal dorsal horn cells, and thereby contribute to the SCS-induced analgesia [64]. Shechter et al. discovered that mechanical hypersensitivity was intensity- and frequency-dependent when compared with high-frequency and conventional methods of spinal cord stimulation in neuropathic pain [65]. The relative safety, cost-effectiveness, and reversibility of SCS have indicated SCS to be an attractive strategy for managing refractory chronic neuropathic pain [66,67,68,69]. Interestingly, a number of systematic reviews, case series, and randomized controlled trials provide evidence regarding the sustained (~24 months) long-term efficacy of combined therapy of SCS and medical treatment [70,71,72,73,74,75,76]. Two clinical trials have reported greater pain relief and enhanced quality of life in painful diabetic neuropathy patients compared with controls [77,78]. Additionally, a weak recommendation was provided by the current European guidelines for the use of SCS (in combination with medical treatment) in neuropathic pain, especially in diabetic neuropathic pain [12,56,79].

The amplitude and time course of attenuating mechanical hypersensitivity were different in both cases, one of which was applied with 50 Hz and the other with 1 kHz. Therefore, it was suggested that the differences in both might be due to the involvement of different peripheral and spinal segmental mechanisms [80,81]. Randomized controlled clinical trials conducted for treating complex regional pain syndrome and chronic lower back syndrome using SCS have proven to be efficacious [82].

In the treatment of neuropathic pain, SCS is used as an effective substitute for local non-surgical therapies. However, SCS is not easy to implement, because it requires the placement of electrodes in direct contact with the spinal cord and necessitates the implantation of electrical hardware. This makes this procedure surgical, which includes significant risks such as infection (4.5% cases) and pain at the generator site (12.0% cases). Other risks include potential nerve damage, bleeding, and epidural punctures [83,84,85]. Proper aseptic procedures, systematic check-ups, and follow-ups can minimize these types of complications [86,87]. Apart from the risk associated with patients, malfunctioning hardware (such as lead and instrumental failure) may be the other factors associated with SCS [88]. Additionally, these types of stimulation devices are usually the last resort and are usually used in patients that have failed conventional medical management.

SCS has the potential to become a revolutionary treatment for chronic neuropathic pain. SCS treatment can minimize chronic pain and helps in improving lifestyles in patients with sciatica, arachnoiditis, and spinal cord injuries [89]. A recently developed wireless SCS system (Stimwave Technologies Inc., Pompano Beach, FL, USA) uses an external battery implanted on the buttock for transmitting the energy. This is very useful for pediatric patients due to its simplified procedure, less implanted hardware, and flexibility in programming [84]. SCS has also been reported to affect the cortical regions of the brain, especially those which regulate motivation and emotions [90]. Therefore, SCS not only helps in the attenuation of neuropathic pain, but may also help in treating co-morbidities such as depression and stress associated with it [91]. However, there is more bias in using SCS for treating neuropathic pain because various waveforms are used, and prospective clinical trials of SCS are required to clinically practice SCS for treating neuropathic pain [92]. Major complications associated with SCS mainly include hardware dysfunctions such as disconnection, migration, and electrode breakage, which potentially require additional surgery [74,93]. Additionally, pain at the implant site may be reported. The risk of damage to the spinal cord, either by direct lesions or indirect compression (epidural hematoma), is extremely low. Nevertheless, the success of SCS in the management of neuropathic pain depends on the proper selection of individuals based on sensory phenotypes, psychological traits, reduced conditioned pain modulation, and augmented central sensitization. Moreover, over the last few decades, SCS has emerged as a potentially valuable treatment; however, the exact mechanisms, including the biochemical and neurophysiological mechanisms, are only partially understood. Preclinical studies for neuropathic pain have only explored the therapeutic mechanisms at spinal and supraspinal level, and extensive clinical studies are also needed to improve their effectiveness because the current SCS computer model still presents significant failure at a rate of 30%. Other components of the pain pathway, including the dorsal horn, are also crucial to be further elucidated with respect to SCS for them to actually be utilized at clinical level for the effective treatment option for neuropathic pain.

#### 7.1.2. Steroid Injection and Neural Blockade

A perineural steroid injection effectively provides transient relief from neuropathic pain, particularly compression-related and trauma-related peripheral neuropathy [94]. In a clinical study, methylprednisolone-mediated peripheral nerve blocking was also found to provide pain relief by effectively reducing abnormal neuronal discharge. Moreover, it was also found to be significantly more effective than lidocaine. Meta-analysis and systemic reviews of epidural steroid administration have demonstrated immediate moderate relief from pain [12,95,96]. However, no significant effects were seen in reducing the risk of undergoing surgery. A weak recommendation has been provided for the use of epidural steroids and local anesthetic nerve blockades to manage acute zoster-associated neuropathic pain and lumbar radiculopathy [2]. Additionally, uncertainties in the actual position of neuronal injury and accuracy of the nerve blockage remain major limitations of steroid injection and neuronal blockage. Additionally, the sympathetic ganglionic blockade has also been used to manage the complex regional pain syndromes, although the long-term benefit is yet to be determined [2].

#### 7.1.3. Transcranial and Epidural Stimulation

**Figure 5:**
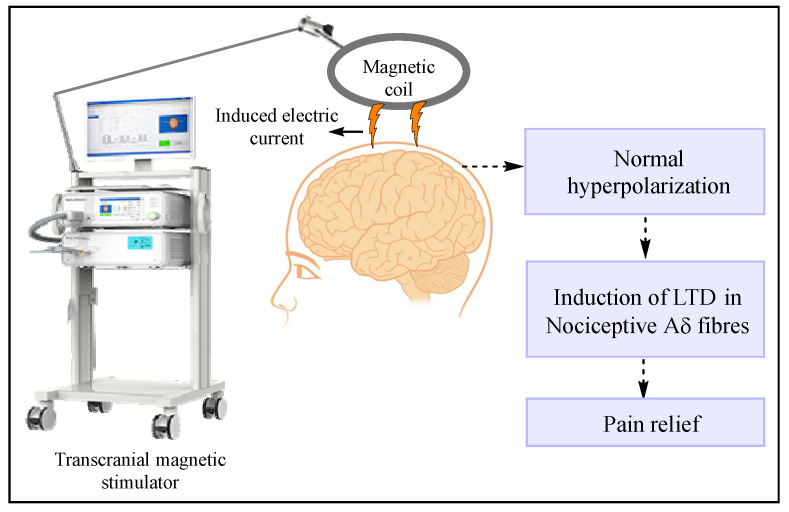
Transcranial magnetic stimulation in the modulation of neuropathic pain. The capacitor is charged at <3 kV, and the circuit is closed via a switch that controls the current flow. An insulated coil placed over the scalp delivers brief magnetic pulses and induces an electric field in the brain to stimulate the nerve cells. Low-frequency (0.3 Hz) repetitive transcranial magnetic stimulation initiates the de novo long-term depression (LTD) of pre-potentiated synapses of nociceptive fibers present in the cortex.

Transcranial magnetic stimulation, or TMS, is a method wherein neural tissues such as the cerebral cortex, spinal nerve root, and peripheral, as well as cranial nerves, are stimulated using a magnetic field [97]. The basic principle involved in TMS is electromagnetic induction, which was discovered by the renowned physicist Michael Faraday in 1838. A schematic representation of TMS in the modulation of neuropathic pain is depicted in Figure 5. While performing a TMS session, a non-conductor coil is placed over the scalp near the head region that delivers brief magnetic pulses and stimulates the nerve cells [98]. TMS can provide either a single pulse of stimulation or repetitive stimuli at various frequencies split up by differing intervals to the affected region [99]. The literature indicates that the application of repetitive electromagnetic induction of 10 Hz on the motor region of the brain exerts an analgesic effect in chronic neuropathic pain patients [100]. This analgesic effect may be attributed to the fact that repetitive electromagnetic induction stimulation may cause the release of striatal dopamine in the human motor cortex and thereby help in pain modulation pathways. However, the exact mechanism of repetitive electromagnetic-induction-mediated pain relief is not known [101].

Two approaches for conditioning repetitive TMS (rTMS) have recently emphasized. One of which is short-duration rTMS with low-intensity and high-frequency stimulation and is called ‘theta-burst’ stimulation, whereas the other is the direct application of weakly negative or constant positive currents on the scalp to enact changes in brain impulses [102]. In a recent study, it was also found that four continuous rTMS sessions can recover refractory central neuropathic pain over three weeks by applying electromagnetic induction of 20 Hz on the primary motor cortex [103]. Therefore, TMS could represent an incredible alternative for treating neuropathic pain. These techniques are convenient because they can be used as portable devices as well. Certain TMS findings will be helpful in the early diagnoses and prognostic predictions of multiple sclerosis, psychogenic paresis, plexus neuropathy, stroke, and cervical spondylosis [104,105,106]. However, rTMS is contraindicated in patients with deep brain electrodes, aneurysm clips, cochlear implants, cardiac pacemakers, and an epilepsy history [2].

Similarly, transcranial direct current stimulation (tDCS) and epidural motor cortex stimulation (EMCS) have also been proposed for the management of refractory cases [107]. tDCS has been found to be beneficial in several peripheral neuropathies [108], whereas a meta-analysis has shown the effectiveness (>40% pain relief) of EMCS in about 60–65% of patients [107]. In general, EMCS requires the precise placement of the stimulating electrode in the motor cortex region. European guidelines provide a weak recommendation for the use of tDCS in peripheral neuropathic pain, and rTMS and EMCS in refractory chronic neuropathic pain [2,74].

#### 7.1.4. Deep Brain Stimulation (DBS)

Long-term intracranial stimulation in the management of neuropathic pain remains debatable. Multiple-site DBS, targeting potential brain regions, including the motor cortex, nucleus accumbens, sensory thalamus, internal capsule, periaqueductal/periventricular grey, septum, anterior cingulate cortex, and posterior hypothalamus, has been assessed to control pain sensations [109,110]. Although the U.K. National Institute for Health and Care Excellence (NICE) guidelines recommend using DBS in refractory patients, recent evidence suggests significant risks of DBS, including wound infections, lead fractures, and intra-operative seizures [111]. In contrast, European guidelines provide inconclusive recommendations for the use of DBS [2,79].

#### 7.1.5. PENS

Percutaneous neuromodulation therapy (PENS) is an innovative and minimally invasive technique used as an interventional therapy to reduce pain hypersensitivity. PENS is an electroanalgesic therapy that amalgamates transcutaneous and electrical nerve stimulation as well as electroacupuncture by placing disposable acupuncture needle-like probes percutaneously to stimulate peripheral sensory nerves innervating the region of neuropathic pain [112]. It is a better alternative for patients who failed to achieve pain relief from transcranial electrical nerve stimulation or electroacupuncture due to the conduction constraints [113]. The term ‘percutaneous neuromodulation therapy’ precisely illustrates the neurophysiologic foundation for PENS-induced analgesia. It includes placing a disposable needle probe (10, 32 gauge) into the soft tissue and/or muscle just below the skin surface close to the specific nerve to electrically stimulate peripheral sensory nerves, innervating the region of neuropathic pain [114]. The treatment plan consists of thirty-minute sessions, either once or twice a week, requiring approximately eight to ten sessions.

Although the detailed mechanism of PENS-induced analgesia is not yet known, it is conjecture that electrical stimulation blocks the transmission of pain signals and releases endorphins and serotonin within the central nervous system [115]. It has also been reported that PENS is highly effective in short-term pain management, because there was a difference in the quality of life of patients who showed improved mood, functionality, and quality of sleep [116]. This can be used in several pain conditions, but not as a replacement for conventional pain medications and only as a supplementary therapy that could decrease the dosage in the extensive regimen.

#### 7.1.6. Neuroablative Procedures and Stimulation of DRG and the Peripheral Nerve or Nerve Field

In addition to the SCS, stimulation of the subcutaneous peripheral nerve field present external to the spinal cord (e.g., DRG and peripheral neurons) has been found to be effective in several forms of chronic neuropathic pain, including PHN and occipital neuralgia [117,118]. A prospective cohort trial found that DRG stimulation reduced pain by 56% in patients with chronic neuropathic pain, with a 60% responder rate (i.e., pain reduction of more than 50%) [119]. Nevertheless, these preliminary findings are undergoing further assessment in controlled clinical trials. Neuroablative procedures such as pulsed radiofrequency (PRF) usually work by the application of an electrical field (or heat bursts) to the targeted site (such as the DRG) without causing any permanent damage to the nerves [119].

Cyroneuroablation is emerging as a novel alternative for the management of pain. Recent advances have also rapidly focused on the painless CRYO-S approach because interventional pain management provides long-term relief from pain (up to 1 year). Cryoneuroablation does not cause permanent damage to neuronal structures, thus allowing them to recover without any risk of neuritis due to procedural inefficacy. Moreover, evidence has associated cryoablation with better outcomes as compared with radiofrequency ablation by an increased rate of atrial contractility and showing better functioning of the left ventricle [120].

Interestingly, novel approaches have aimed to reduce associated adverse effects such as dysesthesia, rebound neuralgia, and sensory loss, while improving the symptoms and durability of pain relief. Recently, a low-temperature PRF ablation or coblation technology was examined for its effectiveness in treating neuropathic pain [121]. This treatment modality utilizes radiofrequency to excite the electrolytes of a conductive medium (e.g., saline) to form energized plasma, which creates radicals that cause tissue dissolution at 40–70 °C. However, coblation may suppress DRG stimulation to downgrade the erroneous ectopic input to the central nervous system, which is the probable mechanism in neuropathic pain relief [122].

In addition, cryoneurolysis, an existing although considerably under-utilized modality that works by freezing the nerves and preventing sensory nerve conduction, has recently been upgraded as an “ultrasound-guided cryoneurolysis modality” for refractory neuropathic pain. A recent study (n = 22) evaluated the efficacy of percutaneous cryoablation in refractory peripheral neuropathic patients [123]. It exhibited a statistically significant reduction among the pre- and post-procedural pain scores and showed no major procedural complications.

### 7.2. Preclinical (or New Experimental) Pain-Controlling Agents

#### Future Directions of Chemogenetics and Optogenetics as Potential Interventional Strategies

Chemogenetics and optogenetics are approaches that have drastically transformed research in the field of neuroscience in the past decade, because they allow us to maneuver anatomically and genetically restricted neurons for assessing physiological or behavioral consequences and could potentially be a highly valuable therapeutic approach in the future [124,125].

**Figure 6:**
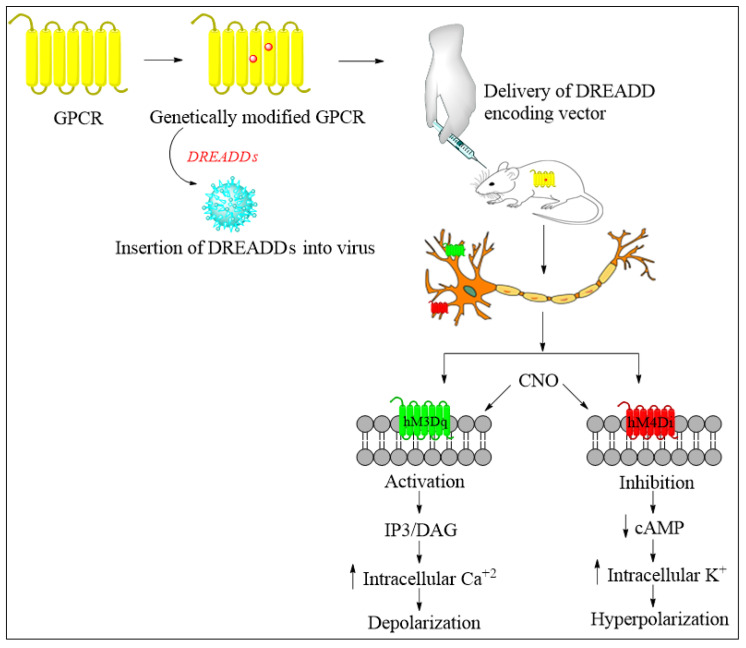
Chemogenetic approach for the silencing of a neural circuit in the brain and spinal cord to treat neuropathic pain. Genetically modified GPCR (DREADD) is developed by a novel directed molecular evolution approach using a biologically inactive metabolite of clozapine, CNO, as the synthetic ligand, and the human M3 receptor (M3R) for initial mutagenesis. This DREADD is inserted into the virus vector. Then, the DREADD encoding vector is inserted into the rat for its expression. CNO binding to hm3Dq and hm4Di leads to neuronal firing and silencing, respectively. Neural silencing of nociceptive signals in the brain and spinal cord by the hyperpolarization of neurons is the approach to treating neuropathic pain. G-protein-coupled receptor, GPCR; designer receptor exclusively activated by designer drugs, DREADDs; clozapine-N-oxide, CNO; human muscarinic receptor 3, hm3Dq; human muscarinic receptor 4, hm4Di; inositol triphosphate, IP3; diacylglycerol, DAG; cyclic adenosine monophosphate, cAMP.

Chemogenetics is a technique in which small molecules are used as a ligand to activate the G-protein-coupled receptors (GPCRs) or generate ionic conductance, eventually affecting neuronal excitability [125]. The vital tool in this technique is designer receptors exclusively activated by designer drugs (DREADDs), wherein ligands such as clozapine-N-oxide (CNO) or perlapine are used to activate the GPCRs (Figure 6) instead of the previous muscarinic acetylcholine receptors that were activated by the endogenous agonist acetylcholine. A recent development is that a new DREADD has been developed in which the receptor is the κ-opioid, whose endogenous ligand is dynorphin A [124,126,127]. Chemogenetics are currently used to decipher the role of glia, especially astrocytes, because they play a crucial role in neuropathic pain. It has also been found that modification of Gi-DREADD signaling in the microglial cells may cause a reduction in neuropathic pain by regulating the pro-inflammatory cytokine levels [128]. Interestingly, it was found that chemogenetic inhibition of the amygdala could terminate comorbid symptoms such as anxiety associated with neuropathic pain [129]. Therefore, chemogenetics might be a good therapeutic approach for treating neuropathic pain in the future. However, further studies are required to obtain insight into implementing chemogenetics as a treatment method for neuropathic pain.

**Figure 7:**
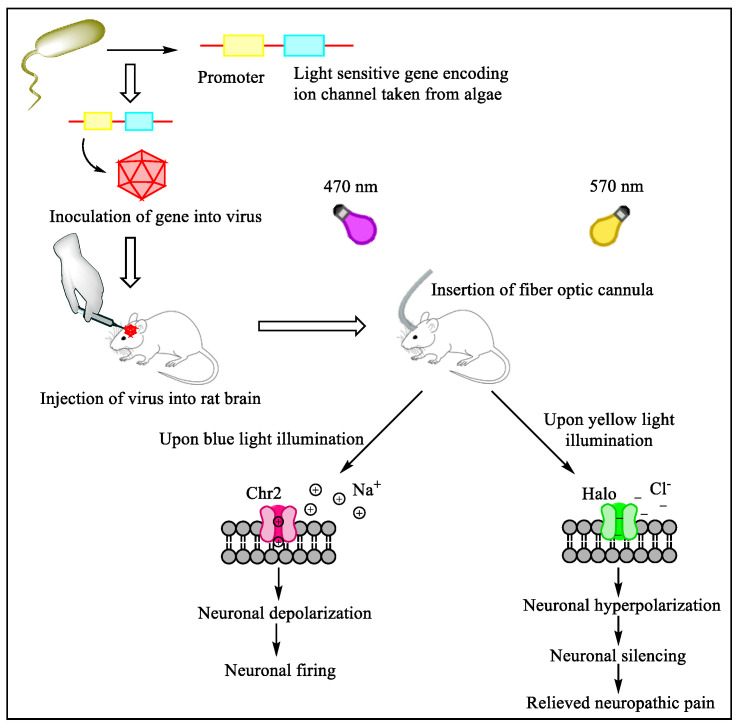
Optogenetic modulation of pain perception. Algal opsin (‘light-sensitive’ gene encoding ion channel) combined with the optogenetic actuator (promoter) is inoculated into a virus. This virus is delivered into the rat brain, where this light-sensitive ion channel expression takes place in neurons upon light illumination at particular wavelengths. At 570 nm, yellow light activates halorhodopsin, which ultimately leads to neuronal silencing and provides relief from neuropathic pain. Channelrhodopsin, Chr2; halorhodopsin, Halo.

Optogenetics is a field that uses light to activate photosensitive targets for modulating neuronal activity [124]. Light of a specific wavelength is used as a stimulus for modifying genes responsible for transmembrane channels, thereby allowing spatial and temporal influence on particular neurons. The system includes an opsin (light-modulated gene or product), a vector for delivering, and a light-delivering instrument (Figure 7). Channels such as light-sensitive halorhodopsin or channelrhodopsin are being explored. A new opsin, named step-function inhibitory channelrhodopsin, has been developed, which increases chloride conductance and is expressed in the unmyelinated primary afferent nociceptors that produce inhibitory responses in mechanical-, thermal-, and formalin-induced nociception. Recently a light-reactive μ opioid receptor (MOR) has been designed to study the spatiotemporal dynamics of MOR signaling, which was upgraded to a higher level when a GPCR chimera receptor was combined with rat rhodopsin RO4 and MOR intracellularly and named opto-MOR. It has been found that optogenetics could reverse mechanical allodynia and hyperalgesia in neuropathic pain mouse models [130,131]. The advantage of using optogenetics is that we can specifically target the neurons and control their activity. However, specific issues arise while applying optogenetics to pain treatment, such as optimizing the opsins to establish a stable neuronal activity that can last longer. Further development of optogenetics into translational research will mark it as a powerful therapeutic approach for treating neuropathic pain [132].

Recent pre-clinical and clinical studies have demonstrated the potential application of chemogenetics and optogenetics by interfering with and manipulating the activity of the neurons [133]. However, one of their limitations is in actually pinpointing the injured nerve fiber and thoroughly understanding the difference between the genetics of damaged neurons to normal functioning neurons, because a small mislocation can result in major hampering of the normal functioning neuronal circuits [134]. Additionally, the major concerns of the gene and AVV delivery system in humans restrict it to emerge as a potential therapeutic approach for neuropathic pain. Thus, future research should focus on non-invasive technologies such as the systemic administration of lipid-based microbubbles and transcranial FUS, resulting in non-invasive neuronal stimulation [135,136]. Although chemogenetics and optogenetics pose serious limitations at the current point, they have the potential to be developed into powerful therapeutic approaches due to their high specificity, thereby probably providing a permanent solution to neuropathic pain.

### 7.3. Gene Therapy against Neuropathic Pain

Gene therapy is a technique which was introduced about three decades ago that helps to correct flawed genes either by replacing them with healthy ones or by adding genes to counteract the disease-causing effect of the defective ones. This helps to increase the expression of specific proteins involved in the formation of receptors, ion channels, neurotransmitters, and biochemical mediators that play a significant role in maintaining physiological conditions in the body. It has several advantages over traditional pharmacological treatments because it can be used to find targets at the genetic level and not merely treat the symptoms of the disease, thus making it comparatively more effective [137]. It also reduces unwanted adverse effects because it is target-specific and not likely to develop tolerance [138]. It can also be combined with other conventional treatment methods.

Gene therapy has enabled us to work with mechanisms that were not previously approachable by pharmacotherapy. Considerable research is being undertaken in this field, and several interesting approaches have been demonstrated so far. Essential molecular targets used in gene therapy for pain relief include spinal opioid gene therapy and anti-inflammatory cytokine gene therapy [139].

### 7.4. Spinal Opioid Gene Therapy

Due to the increased usage of opioid-based drugs in the intervention of neuropathic pain, opioid systems have become an interesting target for gene therapy. The intrathecal delivery of opioids has been sufficient to produce an analgesic effect [140,141]. Therefore, transferring a particular opioid gene using a vector such as the recombinant adeno-associated virus (AAV) into DRG is widely practiced to reduce chronic pain in pre-clinical studies [142,143]. The vector contains complementary DNA of the opioid receptor, which has to be inoculated into the rats’ primary afferent neurons, leading to an upregulation of opioid receptors in the DRG, which may last for up to 6 months [144,145]. Moreover, all these techniques are under preclinical trial, and have not yet been used in humans [146,147].

Two key strategies that developed from spinal opioid gene therapy have proven to be efficacious to induce analgesia in neuropathy in preclinical models. Glorioso and Fink described a novel approach for attenuating pain without the induction of tolerance or systemic side effects by the intradermal inoculation of an HSV vector delivering pain-modulating transgenes to sensory neurons in vivo along with standard pain treatments [148]. The other approach based on opioid delivery included the administration of opioid receptor encoding genes [149]. These approaches were proven to be successful in reducing hyperalgesia and mechanical allodynia in various neuropathic and inflammatory pain models of rodents [150]. This development may eradicate the noxious effect of opioids being administered exogenously, such as the extra-spinal effect seen in peripheral tissues as well as in the central nervous system [151].

#### Anti-Inflammatory Cytokine Gene Therapy

In various animal models of pain, anti-inflammatory cytokines, namely, interleukin (IL)-4, IL-10, and IL-13, were found to exert analgesic effects. IL-10 is an anti-inflammatory cytokine which is involved in the inhibition of pro-inflammatory cytokines; therefore, the delivery of IL-10 encoding genes may help to reduce chronic neuropathic pain. The delivery of IL-10 is performed using various vectors such as HSV and AAV [152]. The expression of IL-10 was able to attenuate allodynia as well as hyperalgesia, a finding which demonstrated that insertion of the AAV vector might lead to overexpression of the IL-10 receptor [153].

Tumor necrosis factor-alpha (TNF-α) is one of the key cytokines involved in the progression of chronic neuropathic pain-like states. However, the activity of TNF-α can be suppressed using treatment with IL-10. Guedon et al. demonstrated that the downregulation of TNF-α was observed in a chronic constrictive injury (CCI)-induced neuropathic pain model by administering IL-10 intrathecally through the AAV vector [139]. IL-10-based gene therapy was found to be effective in varicella-zoster virus-induced pain because it reduced the allodynia [139,154]. IL-4 is another important anti-inflammatory cytokine whose role is being investigated in different models of pain. Treatment with IL-4 has shown significant results in pain associated with diabetic neuropathy and the spinal nerve ligation model of neuropathic pain [155,156].

### 7.5. Ion Channel Targeting

It is a well-established fact that the process of pain transmission and signaling depends upon ion channels, which can thus be considered a primary target in treating conditions such as chronic neuropathic pain. Voltage-gated channels or leak channels could regulate the resting membrane potential [157,158]. Damage, nerve lesion, or inflammation could result in hyper-responsiveness of the ion channels, thus leading to unregulated neuronal firing [159]. Modulation of these ion channels should be studied for possible analgesic effects. This review focuses on recent developments of various ion channels that determine hyperexcitability of the sensory fiber and are emerging as promising targets for the treatment of pain. Currently, certain venom peptides are also being studied for their ability to modify ion channels and are believed to be a powerful therapeutic approach for treating complex neurological disorders in the future [160].

#### 7.5.1. Sodium Channel Modulation

Generation of the action potential in any excitable cell, even in neurons, could be triggered by sodium channels [161]. Neuronal excitability and synaptic plasticity are the central mechanisms involved in pain; therefore, targeting these channels could be a useful approach [162]. Nine types of sodium channels have been identified that have a similar overall structure with a few minor differences in the sequence of amino acids (Na_v1_.1 to Na_v1_.9) [158]. Among these, subtypes Na_v_1.3, Na_v_1.6, Na_v_1.7, Na_v_1.8, and Na_v_1.9 were found to have a role in nociception, because they were upregulated in several pain models [157].

In many patients, pain disorders are inherited due to mutations in genes that encode voltage-gated sodium channels. In pre-clinical studies, Na_v_1.6, which is present at nodes of Ranvier has been found to be essential for nociception. Knockout of Na_v_1.6 in mice has been found to completely ameliorate the painful condition. Additionally, because Na_v_1.6 excites repetitive neuronal firing, which causes pain transmission, its inhibition via siRNA significantly reduces pain along with local inflammation. Moreover, high expressions of Na_v_1.6 have been actively reported in bursting cells of inflamed DRG. These mutations either lead to a gain or loss of function. Modification in the Na_v_1.7 subtype was found in a condition called erythromelalgia, which is manifested by extreme heat hyperalgesia [161,163], whereas Na_v_1.8 and Na_v_1.9 were associated with small-fiber neuropathy [164]. Adverse effects such as double vision, delirium, and somnolence are major limitations of the available non-specific sodium channel blockers; therefore, it is essential to develop new molecules [165]. A few molecules under clinical trials are being investigated for their anti-hyperalgesic properties, including PF-04531083 (Na_v_1.8 blocker, Pfizer, New York, NY, USA) for diabetic neuropathy; CNV1014802, also known as raxatrigine (Na_v_1.7 blocker, Convergence,) for trigeminal neuralgia and lumbosacral radiculopathy; and XEN402 (funapide) (Na_v_1.7 blocker, Xenon, and Teva Pharmaceuticals) for inherited erythromelalgia and postherpetic neuralgia [164]. Among various voltage-gated sodium channels, Na_v_1.7 is considered a particularly promising target because congenital pain insensitivity is due to the mutation occurring in Na_v_1.7. However, translation of these studies is difficult because animal models do not show complex pain states, and pain cannot be terminated by a single target because it is not unifactorial. Continuing research in this field might help further the development of novel potent analgesics [166].

#### 7.5.2. Calcium Channel Modulation

Voltage-gated calcium channels play a major role in the conduction of pain signals in nociceptive neurons at the spinal level [167]. Three main types of channels are recognized, which are categorized as Ca_v_1, Ca_v_2, and Ca_v_3. Overexpression of Ca_v_2.2 in the outermost surface of the dorsal horn, which is considered to be the nociceptive region of the spinal cord, leads to neuropathic pain [168]. N-type calcium channels in the DRG neurons are involved in pain signaling, which could be studied to establish new targets [169]. N-type channels play an important role in developing pain, whose activation can be inhibited by suppressing the release of substance P or using MOR agonists, such as morphine, which block these channels [170]. The most popular drug in clinical practice, gabapentin [171], acts upon the α2-δ subunit of N-type calcium channel and reduces the release of calcium in DRG neurons and synaptosomes, thereby producing an effective analgesic action in neuropathic pain patients, particularly with conditions such as trigeminal neuralgia, diabetic neuropathy, PHN, and migraine [172]. The U.S. FDA has approved a potent N-type Ca_v_ antagonist, Prialt (Elan Pharmaceuticals, San Diego, CA, USA), with a generic name of Ziconotide, also identified as omega conotoxin MVIIA. It is a 25-amino-acid disulfide-bridged polypeptide derived from the venom of *Conus magus* [173]. In a recent study, it has also been found that the natural compound physalin F was able to block the voltage-gated calcium channels, and also reduced pain hypersensitivity in peripheral neuropathy induced by paclitaxel [174]. Hence, targeting voltage-gated calcium channels might be a good approach for discovering novel analgesics to treat neuropathic pain.

#### 7.5.3. Potassium and Chloride Channel Modulation

Potassium channels have a crucial role in maintaining neuronal excitability because they cause hyperpolarization, which inhibits continuous depolarization that causes the hyperexcitability of neurons. Downregulation of potassium channels is observed in nociceptive signaling. There are several potassium channels, among which the K_v_1.2 subtype has been studied thoroughly [40]. Fan et al. suggested that K_v_1.2 expression was downregulated in a time-dependent manner in DRG neurons following L5 spinal nerve ligation and sciatic nerve axotomy models of neuropathic pain [175]. They further reported that downregulation was rescued by the overexpression of DRG K_v_1.2 RNA upon the injection of AAV5-K_v_1.2 viral particles [176]. There is a predominant expression of different potassium channel subunits in different neurons, such as K_v_1.1 and K_v_1.2 in myelinated sensory axons and K_v_1.4 in C-fibers [177]. In another study, it was found that K_v_1.2 expression was downregulated in the CCI model of neuropathic pain, and the use of an miR137 antagonist reversed this condition and resulted in attenuation of the nociceptive response [177]. K_v_7 channels are also being targeted because they can suppress the hyperexcitability of neurons. Researchers have shown special interest in the K_v_7 channels, because their activators can help to reduce neuronal hyperexcitability and attenuate pain. Retigabine, a K_v_7 activator, was able to ameliorate cold hyperalgesia and hypersensitivity of a damaged paw in the CCI model of neuropathic pain. However, the use of retigabine in treating seizures has been stopped due to its undesired effects. Further studies on the K_v_7 channel are required before suggesting the clinical use of K_v_7 activators to treat neuropathic pain [178]. Regulating K_v_ function through the modulation of silent subunits may be an interesting approach to treatment [165]. To date, no analgesic drugs modulate pain signaling through potassium channels.

In addition to potassium ion channels, the dysregulation of chloride ion channels has been found to be associated with increased levels of pain hypersensitivity. Restoring the GABA-mediated inhibition in nerve injury models of mice by KCC2 blockade has also been shown to give rise to allodynia and the increased spiking of neuronal firing, causing pain behaviors in animals. Furthermore, the differential homeostasis of chloride ions in rodent spinal dorsal horns has been found to generate sensitization via spontaneous neuronal firing, initiating pain.

## 8. Conclusions and Prospects

Neuropathic pain is a burden to clinicians and patients due to a lack of development in treatment options. The contemporary treatments used in clinical settings exert several serious side effects, which limit their use in patients. Interventional strategies such as spinal cord stimulation are used clinically and have shown good results in attenuating pain responses. TMS was also able to attenuate pain and treat comorbid conditions due to its ability to control the brain’s cortical regions. Other techniques, such as optogenetics, chemogenetics, gene therapy, PENS, and targeting ion channels such as voltage-gated sodium, calcium, and potassium channels, have also shown interesting results in alleviating neuropathic pain. However, most of these techniques are still in the preclinical stage and face difficulties in translation to clinical studies due to limitations in the currently available animal models. Continuing research on these interventional strategies will further the development of promising novel therapies that can improve the quality of life in patients suffering from neuropathic pain. Undoubtedly, neuromodulation is now considered a cutting-edge treatment modality due to the repeated failure of pharmacological interventions or their associated adverse effects. Thus, neuromodulation seems to represent the future of neuropathic pain management. However, interventional strategies must extend beyond the nerve signal blockade and the regulation of signal transmission, modulation, and processing in order to contribute to ensuring the longevity of therapy. Continuing efforts towards the development of novel interventional methods in the management of neuropathic pain are evident; thus, it can be inferred that the future lies in two major domains: firstly, interventions possessing multimodal mechanisms, and secondly, interventions offering synergistic longevity in therapy with no development of tolerance. Certainly, as the burden of neuropathic pain continues to increase, there is an urgent need to investigate and develop novel multimodal interventions.

## Figures and Tables

**Table 1:** Current pharmacotherapies used in neuropathic pain treatment.

Class of Drug	Nature of Action	Undesired Effects	Contraindication	Reference
Tricyclic antidepressants (Nortriptyline, Desipramine)	Inhibition of serotonin as well as norepinephrine reuptake, sodium channel blockade, anticholinergic	Sleeplessness, anticholinergic effects (e.g., xerostomia or ischuria, obesity)	Cardiovascular disease, epilepsy, glaucoma	[43]
Serotonin–norepinephrine reuptake inhibitors (Duloxetine, Venlafaxine)	Inhibition of both serotonin and norepinephrine reuptake	Nausea	Hepatic malfunction, impaired renal function, alcohol use disorder	[45]
Calcium channel α2-δ ligands (Gabapentin, Pregabalin)	Diminishes the release of glutamate, norepinephrine as well as substance P, with α2-δ subunit ligands of voltage-gated calcium channel	Somnolence, lightheadedness, peripheral edema	Impaired renal function	[55]
Opioid agonist (Morphine)	Agonist action on μ-receptor	Nausea, emesis, improper bowel movement, vertigo	History of substance abuse increased suicidal risk	[51]

## Data Availability

Data sharing is not applicable.
